# MANF Is Essential for Neurite Extension and Neuronal Migration in the Developing Cortex

**DOI:** 10.1523/ENEURO.0214-17.2017

**Published:** 2017-09-29

**Authors:** Kuan-Yin Tseng, Tatiana Danilova, Andrii Domanskyi, Mart Saarma, Maria Lindahl, Mikko Airavaara

**Affiliations:** 1 Program in Developmental Biology, Institute of Biotechnology, University of Helsinki, Helsinki, 00014, Finland; 2 Department of Neurological Surgery, Tri-Service General Hospital and National Defense Medical Center, Taipei, Taiwan

**Keywords:** cerebral dopamine neurotrophic factor, ER stress, MANF, neurotrophic factor, UPR

## Abstract

Mesencephalic astrocyte-derived neurotrophic factor (MANF) is an endoplasmic reticulum (ER) resident protein with neuroprotective effects. Previous studies have shown that MANF expression is altered in the developing rodent cortex in a spatiotemporal manner. However, the role of MANF in mammalian neurogenesis is not known. The aim of this study was to determine the role of MANF in neural stem cell (NSC) proliferation, differentiation, and cerebral cortex development. We found that MANF is highly expressed in neural lineage cells, including NSCs in the developing brain. We discovered that MANF-deficient NSCs in culture are viable and show no defect in proliferation. However, MANF-deficient cells have deficits in neurite extension upon neuronal differentiation. *In vivo*, MANF removal leads to slower neuronal migration and impaired neurite outgrowth. *In vitro*, mechanistic studies indicate that impaired neurite growth is preceded by reduced *de novo* protein synthesis and constitutively activated unfolded protein response (UPR) pathways. This study is the first to demonstrate that MANF is a novel and critical regulator of neurite growth and neuronal migration in mammalian cortical development.

## Visual Abstract

**Figure:**
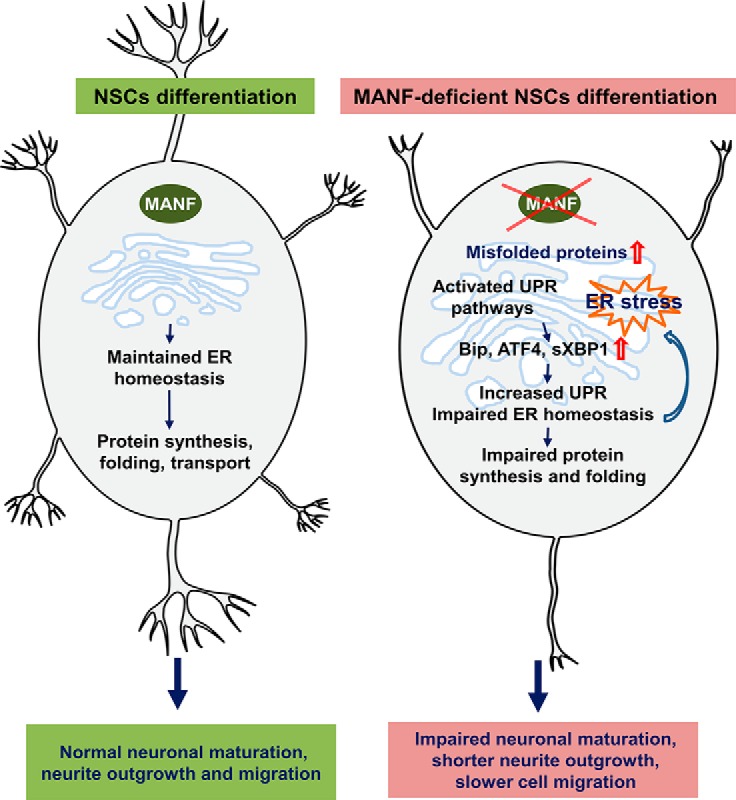

## Significance Statement

Mesencephalic astrocyte-derived neurotrophic factor (MANF) is a neuroprotective protein, and its expression is high in the developing cortex. However, its role in mammalian neurogenesis is unknown. Here we show that MANF is highly expressed in neural lineage cells, including neural stem cells (NSCs) of the subventricular zone (SVZ) in the developing mouse brain. Unlike other neurotrophic factors, removal of MANF does not alter stem cell proliferation. However, removal of MANF disrupts neurite growth and migration of developing neurons into the cortex. Data in the current manuscript suggests that endogenous MANF is involved in neurite extension, as removal of MANF in NSCs leads to reduced *de novo* protein synthesis and activated unfolded protein response (UPR) on differentiation.

## Introduction

The neocortex is composed of various cell types having distinct morphology, position, pattern, and physiologic properties (Sato et al., 2012; Woodworth et al., 2012; Greig et al., 2013). Differentiation of neural stem cells (NSCs) or neural progenitor cells (NPCs) into distinct neuronal subtypes and their migration (Greig et al., 2013) require spatiotemporal activation of complex molecular cascades and proteostasis. Developmental studies in the mammalian neocortex have shown that intrinsic factors, including specific transcription factors, are necessary for cell type specification and differentiation (Grove and Fukuchi-Shimogori, 2003; Molyneaux et al., 2007; O'Leary and Sahara, 2008). The transcription factors Tbr1, CTIP2, and Cux1, expressed in different layers of the developing cortex, contribute to laminar fate determination (Alcamo et al., 2008; Chen et al., 2008; Cubelos et al., 2008; Bedogni et al., 2010). Moreover, extrinsic factors, such as glial cell line-derived neurotrophic factor and brain-derived neurotrophic factor, also regulate proliferation, cell type specification, differentiation, and migration of cortical neurons (Ferri and Levitt, 1995; McAllister et al., 1995; Canty et al., 2009). Previously, mesencephalic astrocyte-derived neurotrophic factor (MANF) and cerebral dopamine neurotrophic factor were identified as a new family of neurotrophic factors (Petrova et al., 2003; Lindholm et al., 2007) protecting dopamine neurons in animal models of Parkinson’s disease (Voutilainen et al., 2009; Airavaara et al., 2012). We have shown that MANF is neuroprotective against cortical neurons in transient ischemic brain injury (Airavaara et al., 2009; Airavaara et al., 2010). Furthermore, we have recently shown that intracellular MANF protects primary neurons *in vitro* only when localized to the endoplasmic reticulum (ER; Hellman et al., 2011; Mätlik et al., 2015). In mouse brain, high *Manf* mRNA levels are detected in the cerebral cortex, hippocampus, and cerebellum (Lindholm et al., 2008). In rat brain, MANF is developmentally regulated in the cortex, where high levels are present in early postnatal days, and its expression declines as the cortex mature (Wang et al., 2014). These findings indicate that MANF is spatiotemporally expressed in the cortex and suggest that it may play a role in the maturation of cortical neurons. However, the brain phenotypes of MANF knockout mice and how deletion of MANF affects neurogenesis in the developing cerebral cortex have not been investigated.

In this study, we aimed to investigate the causal relationship between mammalian cortical neurogenesis and ER homeostasis during neuronal differentiation using MANF-deficient mice as a model system (Lindahl et al., 2014). These studies are necessary because mechanism of action for MANF is unsolved and its receptor(s) unknown. Furthermore, we used MANF-deficient mice to validate the specificity of MANF antibody to study MANF protein localization in the developing brain. We investigated the role of MANF in neurogenesis, neuronal differentiation, neurite growth and neuronal migration. In this report, we provide experimental evidence for a new functional role for MANF in the developing mammalian brain. Mechanistic *in vitro* studies show role of MANF in *de novo* protein synthesis and activation of unfolded protein response (UPR) during neuronal differentiation. Our results suggest that MANF is a crucial factor regulating ER homeostasis in neurons to support neurite growth and subsequent neuronal migration in the development of the cortex.

## Materials and Methods

### Animals

The generation of MANF knockout mice (*Manf^-/-^*) was as described previously (Lindahl et al., 2014). Briefly, *Manf^-/-^* mice are complete conventional knockout mice lacking MANF mRNA and protein in all tissues through efficient splicing of exon 2 to a reporter gene (Lindahl et al., 2014). The day of vaginal plug was designated as embryonic day (E)0.5. All experimental procedures were performed according to the 3R principles of the EU directive 2010/63/EU on the care and use of experimental animals, and local laws and regulations [Finnish Act on the Protection of Animals Used for Scientific or Educational Purposes (497/2013) and Government Decree on the Protection of Animals Used for Scientific or Educational Purposes (564/2013)].

### Isolation and cultivation of embryonic *Manf^-/-^* and *Manf^+/+^* NSCs

The telencephalons along with the lateral ventricle of E13.5 wild-type (WT) and *Manf^-/-^* mice were isolated and triturated in Hanks’ balanced salt solution (Invitrogen) by mild pipetting using 1-ml pipette tips. Dissociated cells were cultured in NSC growth medium containing DMEM/F12 (Gibco) supplemented with B27 (20 µl/ml), epidermal growth factor (EGF; 20 ng/ml), fibroblast growth factor-2 (FGF-2; 20 ng/ml; Gibco), GlutaMAX (10 µl/ml; Gibco), and penicillin-streptomycin (50 U/ml). The viable dissociated cells at a density of 5 × 10^5^ cells/10 ml in NSC growth medium were seeded into uncoated T75 culture flasks at 37°C with 95% atmospheric air/5% CO_2_. Half of the medium was replaced every other day. After 5–7 d of culture, neurospheres were dissociated by trituration and digestion in Accutase solution (Gibco) for 10 min at 37°C, cells were then centrifuged and resuspended in NSC growth medium. Neurospheres were passaged every 7-10 d. Viable NSC numbers were analyzed by staining with 0.5% trypan blue and counted in a cell counter (Bio-Rad). The primary passage of NSCs was not pure and contained a mixed cell population. After passage 10, cultured NSCs start to show signs of reduced viability and differentiation. We used NSCs from passages 3 to 10.

### Self-renewal assay

To evaluate the self-renewal capacity of the cultured NSCs, we used a clonal colony-forming to measure the proportion of cells able to make neurospheres (Kerosuo et al., 2010). Single cell-dissociated cultures of NSCs were plated into 96-well plates with 10-15 cells per well, and the number of formed neurospheres was counted after 7 d. The number of neurospheres (>20 cells tightly attached to each other) was divided by the number of initially plated cells giving the percentage of self-renewing cells.

### BrdU cell proliferation assay

To measure proliferation, the percentage of NSCs in S-phase, NSCs were incubated with BrdU (1 mM, 5-bromo-2'-deoxyuridine; Sigma-Aldrich) for 12–16 h, centrifuged onto glass slides using a cytocentrifuge, fixed, and stained with anti-BrdU antibody. Slides were mounted with VectaShield mounting medium containing 4’-6-diamidino-2-phenylindole (DAPI; Vector Laboratories). A total of 12–18 images of each group was acquired using epifluorescence microscopy and analyzed by Image Pro Plus software (Media Cybernetics). The percentage of proliferating cells was estimated by dividing the number of BrdU-positive cells by the number of DAPI-stained nuclei.

### *In vitro* differentiation and apoptosis assays

To induce differentiation, dissociated NSCs (10^5^ cells/ml) were plated onto cover glasses or four-well plates coated with 0.5 µg/ml laminin/10 µg/ml poly-L-ornithine and cultured in NSC growth medium for 1 d. The next day, the medium was replaced with differentiation medium [penicillin-streptomycin (50 U/ml), B27 supplement (Gibco), 2 mM GlutaMAX (Gibco) in Neurobasal medium (Gibco)], and cells were allowed to differentiate for 7 d (see Figure 3*A*). Recombinant human MANF (rhMANF; 200 ng/ml; Icosagen) was given in the *Manf^-/-^* cells at day *in vitro* (DIV)1. Differentiated cells were immunostained with cell-type-specific antibodies followed by immunofluorescence microscopy. To estimate the number of apoptotic cells after NSC differentiation, cells (10^5^ cells/ml) were fixed with 4% paraformaldehyde (PFA) and labeled for the deoxynucleotidyl transferase dUTP nick end labeling (TUNEL; Roche), according to the manufacturer’s instructions. A near-infrared scanner was used to measure the intensity of the signal at 700 nm (Li-Cor Biosciences, Odyssey). Quantification was performed with Image Studio Software for the Odyssey. The signal of apoptotic cells was quantified from triplicate wells, and each experiment was repeated three to six times.

### Immunofluorescence staining

NSCs grown on glass coverslips were fixed with 4% PFA for 15 min at room temperature (RT) and washed three times with PBS. Cells were blocked in blocking buffer (5% bovine serum albumin, 0.3% Triton X-100 in PBS) for 1 h at RT. Cells were then incubated with the following primary antibodies overnight at 4°C: rabbit anti-MANF (1:400, Icosagen, catatog 310-100), mouse anti-Nestin (1:500; Millipore, catatog MAB353), goat anti-doublecortin (DCX; 1:200 Santa Cruz Biotechnology, catatog sc-8066), rabbit anti-β-tubulin III (Tuj1; 1:1000, BioLegend, catatog 802001), mouse anti-GFAP (1:1000, Millipore, catalog MAB360), or rabbit anti-MAP2 (1:500, Millipore, catatog AB5622). For fluorescence microscopy, appropriate secondary antibodies conjugated with Alexa Fluor 488 or 568, (1:500, Life Technologies) were used. Finally, coverslips were washed three times in PBS, stained with DAPI (Sigma-Aldrich) 5 µg/ml in PBS and mounted in fluorescent mounting medium (SHANDON, ThermoFisher Scientific). Fluorescence images were captured with Zeiss AxioImager M2 482 epifluorescence microscope equipped with a 483 AxioCam HRm camera. Images were acquired with the AxioVision4 software. The percentages of DCX-, Tuj1-, MAP2-, and GFAP-positive cells were determined as the ratio of positive cell numbers to DAPI-stained nuclei from four different visual fields randomly selected on each coverslip. *In vitro* differentiation experiments were performed and analyzed in quadruplicate.

## Immunoblot analysis

Cells were homogenized in NP-40 lysis buffer (50 mM Tris-HCl, pH 7.4, 0.15 M NaCl, 1.0 mM EDTA, and 1% NP-40) freshly supplemented with phosphatase and protease inhibitor cocktail (Complete, Mini, EDTA-free, Roche Life Science), incubated on ice for 15-30 min and centrifuged at 13,000 rpm for 10 min. Proteins were separated by 8% or 15% SDS-PAGE and transferred to nitrocellulose membranes. Immunoblot analysis using antibodies anti-Nestin (1:500, Millipore, catatog MAB353), anti-DCX (1:500, Santa Cruz Biotechnology, catatog sc-8066), anti-TuJ1 (1:1000), anti-GFAP (1:1000, Millipore, catatog MAB360), anti-MAP2 (1:500, Millipore, catatog AB5622), anti-neurofilament (1:750, NF-200, Sigma-Aldrich, catatog N0142), anti-EIF2α (1:1000, Cell Signal Technology, catatog 9721), anti-phospho (p)-EIF2α (1:1000, Cell Signal Technology, catatog 9722S), anti-α-tubulin (1:2000, LI-COR, catatog 926-42213), and anti-GAPDH (1:2000, Millipore, catatog MAB374) was conducted. The membranes were washed twice for 10 min each in PBS containing 0.1% Tween (PBS-T) then probed with goat anti-mouse or goat anti-rabbit IR-Dye 670 or 800 secondary antibodies (LI-COR Biotechnology) in milk blocking buffer (5%) with 0.1% Tween for 1 h at RT. After labeling with secondary antibodies, washes were repeated twice for 10 min with PBS-T, and then membranes were placed in water. Membranes were imaged using a LI-COR Odyssey scanner (LI-COR).

### Analysis of neurite growth

Images of 20–50 neurons from three microscope slides from each experiment were taken with a Zeiss AxioImager M2 482 epifluorescence microscope. Representative cells with strong TUJ1 immunoreactivity in the neurite processes were analyzed. Neurites with lengths of at least twice the diameter of the cell body were measured. Neurite lengths from the soma were traced and measured using ImageJ software.

### RNA isolation, reverse transcription, and qPCR

RNA from NSCs and differentiated cells at each time point were isolated using TriReagent (Molecular Research Center). cDNA from 2 µg of total RNA was produced (RevertAid Premium Reverse Transcriptase, Thermo Fisher Scientific.; dNTP mix, ThermoFisher Scientific; oligo(dT)_15_ Primer, 500 µg/ml, Promega). qPCR was performed using LightCycler 480 SYBR Green I Master mix (Roche Diagnostics GmbH) and Roche LightCycler 480. Relative gene expression levels were normalized to β-actin in each sample. Primers used in qPCR for *Grp78*, Xbp1t, *Xbp1s*, *Atf4*, and *Chop* are published sequences (Lindahl et al., 2014).

### Histology and immunohistochemistry

Whole mouse embryos or isolated brains from E13.5 to postnatal day (P)7 were fixed in 4% PFA at 4°C for at least 3 d and then embedded in paraffin and sectioned. Five-micrometer coronal sections were cut from the cerebral plates (future cerebral cortex) along the anterior horn of the lateral ventricle from *Manf^-/-^* mice and their WT littermates. Slides were deparaffinized and hydrated, then subjected to antigen retrieval in 10 mM sodium citrate buffer, pH 6.0, or 0.05% citraconic anhydride buffer, pH 7.4, at 120°C for 10 min. Immunohistochemistry was performed using the following antibodies: rabbit anti-MANF (1:1000, Icosagen, catatog 310-100), goat anti-DCX (1:400, Santa Cruz Biotechnology, catatog sc-8066), rabbit anti-Tbr1 (Abcam, 1:500, catatog ab31940), rat anti-CTIP2 (1:400, Abcam, catatog ab18465), rabbit anti-CUX1 (Millipore, 1:100), rabbit anti-PAX6 (1:100, Abcam, catatog ab5790), rabbit anti-Tbr2 (1:500, Abcam, catatog ab75720), rabbit anti-phospho-histone H3 (pH3; 1:200, Millipore, catatog 06-570), mouse anti-Nestin (1:400, Millipore, catatog MAB353), mouse anti-NeuN (1:400, Millipore, catatog MAB377), mouse anti-GFAP (1:500, Millipore, catalog MAB 360), mouse anti-NF-200 (1:1000, Sigma-Aldich, catatog N0142), rabbit anti-MAP2 (1:400, Millipore, catatog AB5622), rabbit anti-Reelin (1:300, Santa Cruz Biotechnology, catatog sc-5578), and rabbit anti-activated caspase III (1:200, Cell Signaling, catatog 9664) at 4°C overnight. For fluorescence microscopy, appropriate secondary antibodies conjugated with Alexa Fluor 488 or 568 (1:500; Invitrogen) were used for visualization. For detection of a signal by light microscopy, biotinylated secondary antibody and peroxidase-conjugated streptavidin Vectastain ABC-detection system (Vector Laboratories) were used. Sections were developed with Vector diaminobenzidine peroxidase substrate kit (Vector Laboratories). Cortical layers were defined with cresyl violet staining.

### *In vivo* BrdU labeling and analysis

For the cell division experiment, pregnant *Manf^+/-^* female mice (at E13.5 and E15.5) or their progeny (P1) were injected intraperitoneally with BrdU (50 mg/kg), which was dissolved in sterilized saline solution at a concentration of 10 mg/ml. The injections were given three times with 3-h intervals, and brains were harvested 30 min after the last injection. For the birth-dating study, BrdU (100 mg/kg) was given in a single intraperitoneal injection to a pregnant mouse at E13.5 or E15.5, and embryos were dissected 4 d later (E17 or E19, respectively). The paraffin-embedded brain sections were dewaxed in xylene, rehydrated, processed for antigen retrieval with 10 mM citrate buffer (pH 6.0) and then immunostained with a mouse anti-BrdU (1:200, Abcam, catatog ab1893) overnight at 4°C followed by secondary antibodies conjugated to Alexa Fluor 488 (1:500; Invitrogen) for 2 h at RT. For S-phase progenitor cell quantification, the number of BrdU+ cells in the subventricular zone (SVZ) was counted at E13.5, E15.5, and P1 in each field under 10× magnification. For the birth-dating study of radial migration at E17, the distribution of BrdU-labeled cells was analyzed in the ventricular zone (VZ)/SVZ, intermediate zone (IZ), subplate (SP), and cortical plate (CP), respectively. At E19, to quantify the distribution of BrdU-positive nuclei, the CP from the pial side to ventricular side was divided into SVZ, IZ, cortical Layer V/VI, cortical Layer IV, and cortical Layer II/III.

### Homopropargylglycine (HPG) labeling and click chemistry

For detecting and characterizing newly synthesized proteins during neuronal differentiation, we optimized the protocols for HPG incorporation and fluorescence detection. Dissociated NSCs (10^5^ cells/ml) were plated onto 96-well plates coated with 0.5 µg/ml laminin/10 µg/ml poly-L-ornithine and cultured in NSC growth medium for 1 d. The next day, the medium was replaced with differentiation medium. At times indicated, the medium was removed and replaced with L-methionine-free DMEM (Sigma-Aldrich) for 45 min to deplete methionine before the addition of HPG (Invitrogen) at a final concentration of 50 µM for 1 h in L-methionine-free DMEM. The samples were fixed with 4% PFA for 15 min and subjected to Click-iT reaction cocktail, which contains 10 µM Alexa Fluor 488-azide, 1 mM CuSO_4_, 1× Click-iT HPG reaction buffer, and 1× Click-iT HPG buffer additive. The reaction was allowed to proceed for 40 min at RT in the dark. After removal of reaction cocktail, cells were washed in Click-iT reaction rinse buffer and then stained with DAPI. Fluorescence images were captured with Cellomics CellInsight (Thermo Scientific) equipped with Olympus LUCPlanFL N 20×/0.45 objectives. Images were analyzed with the CellProfiler.

### Imaging and the analysis of imaging data

For bright field imaging, a 3D-Histech (http://www.biocenter.helsinki.fi/bi/histoscanner/index.html) whole slide scanner was used. Image analysis was performed with Image-Pro Analyzer 7.0. Fluorescence or light microscopy images were captured with an Olympus BX61 light microscope equipped with a high-resolution color digital camera and Cell Life Science Microscopy software (Olympus Soft Imaging Solution) or using a Zeiss AxioImager M2 482 epifluorescence microscope equipped with a 483 AxioCam HRm camera. Images were acquired with the AxioVision4 software. Cell-IQ was used to take the videos.

Cell counts in the dorsolateral neocortex were done as previously described (Fukumitsu et al., 2006). The cells were analyzed from an area of dorsomedial neocortical wall above the medial part of the lateral ventricle, which corresponds to the future primary somatosensory representation. Six to 12 coronal sections of the somatosensory cortex from three to six animals were analyzed. After immunofluorescent staining, cells were counted from images obtained by epifluorescence microscopy in a cortical sector ranging from the pial surface to lateral ventricle, 450 × 350 µm at E13.5 and E15.5; 1000 × 600 µm at E18.5, E19, and postnatal age. The number of mice at each time point was at least four per genotype. Four different brain sections per mouse were analyzed. For fluorescent intensity measuring, 8-bit grayscale images were taken at 10× magnification. Mean fluorescence values were measured in the CP at E19 or cerebral cortex at P7 and after background subtraction, the values for *Manf^-/-^* mice were normalized to those of age-matched WT controls.

### Statistical analysis

All graphs and statistic calculations were performed in GraphPad Prism 6.0. A Student’s *t* test or ANOVA followed by Bonferroni correction test was conducted to test statistical significance. All data were tested for normality. All values and graphs in all figures are shown as mean ± SEM. The results with *p* < 0.05 were considered statistically significant.

## Results

### Cell types expressing MANF in the developing mouse cortex

**Figure 1.**
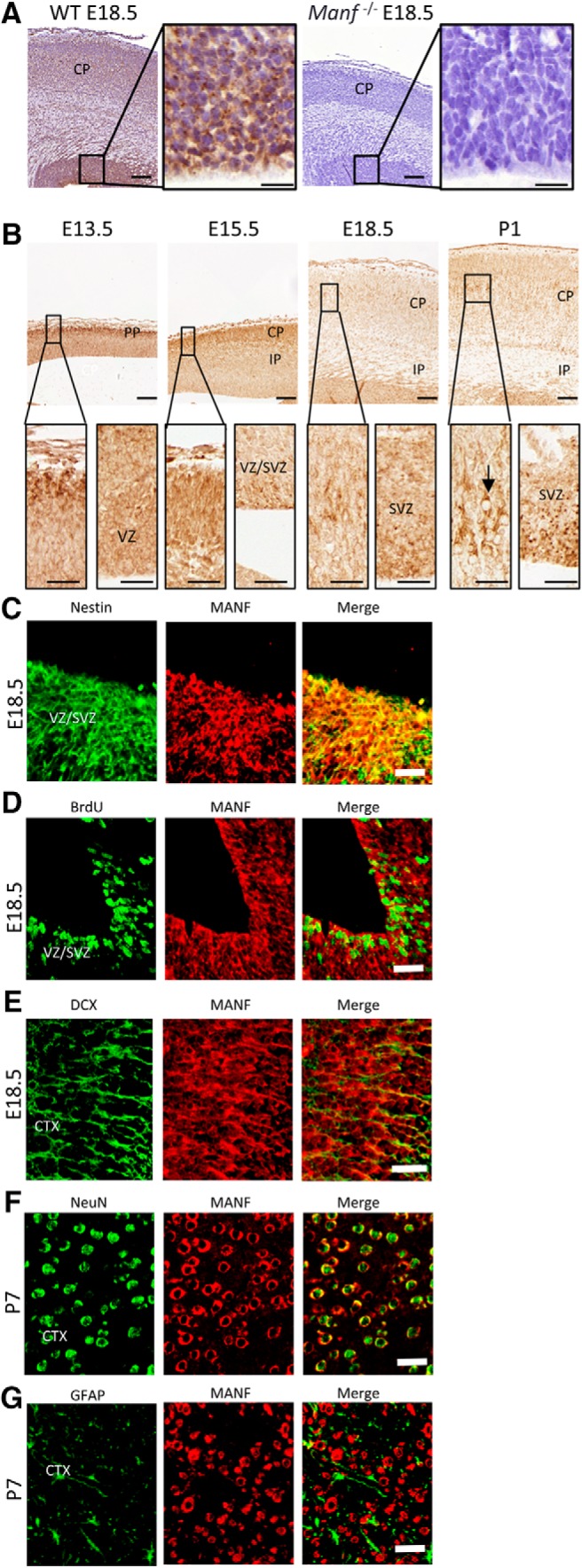
MANF expression in the developing cerebral cortex. ***A***, Coronal cortical sections from E18.5 WT and *Manf^-/-^* brains stained with MANF antibody. The insets show photomicrographs of the VZ/SVZ in higher magnification. ***B***, In the E13.5 and E15.5 brain MANF is predominantly localized in cells of the PP/CP and modestly localized in the VZ/SVZ. By E18.5, strong MANF expression is seen in the VZ/SVZ and CP (inset). At P1, MANF is highly expressed in the VZ/SVZ and middle layers of the CP (inset). At E18.5, VZ/SVZ was double-stained for Nestin (green) and MANF (red) antibodies (***C***) and for BrdU (green) and MANF (red) antibodies (***D***). ***E***, Section of cerebral cortex from E18.5 mice double-stained with DCX (green) and MANF (red) antibodies. ***F***, Section of cerebral cortex from P7 mice double-stained with NeuN (green) and MANF (red) antibodies. ***G***, Section of cerebral cortex from P7 mice double-stained with GFAP (green) and MANF (red) antibodies; scale bars: 100 µm (***A***, ***B***), 20 µm (***A***, ***B*** inset), 50 µm (***C–G***). PP, preplate; CP, cerebral plate; IP, intermediate plate.

MANF mRNA and protein are found in neurons in the developing and adult rodent brain (Lindholm et al., 2008; Wang et al., 2014). Cortical neurons express MANF in Layers II–VI in adult mouse brain and the neopallidal cortex in the E12.5 embryo (Lindholm et al., 2008). We analyzed the expression of MANF by immunohistochemistry during the development of the mouse cortex from E13.5 to P7. The specificity of MANF antibody was validated by comparing WT and *Manf^-/-^* cortical brain sections. MANF immunoreactivity was not observed in *Manf^-/-^* mice with the antibodies used in this study (Fig. 1*A*). We observed high levels of MANF immunoreactivity in the CP of the E13.5 and E15.5 mouse brain (Fig. 1*B*). At E18.5, when the laminated structure of the cerebral cortex develops, higher immunoreactivity of MANF was found in the middle layer of the CP (Fig. 1*B*, insets) compared to other layers. At P1, while MANF was expressed in all layers of the cortex, neurons at Layers IV/V of the cortex showed stronger MANF immunoreactivity (Fig. 1*B*, inset, arrow). Interestingly, MANF protein was preserved in cells in the neurogenic areas, VZ and SVZ, at all time points (Fig. 1*B*). To identify cells expressing MANF in the developing brain, we used double immunofluorescent staining with antibodies against Nestin and DCX, which are widely used markers for NSCs and NPCs, respectively. BrdU was used to label S-phase progenitor cells. MANF was colocalized with Nestin (Fig. 1*C*) and MANF was found in BrdU-positive cells (Fig. 1*D*) in VZ/SVZ of E18.5 brain. Also, MANF was found in DCX-positive cells at E18.5 (Fig. 1*E*) and in NeuN-positive cells, but not in GFAP-positive cells at P7 (Fig. 1*F*,*G*) in mouse cortex. These results demonstrated that MANF is not only expressed in postmitotic neurons but also in mitotic NSCs in the developing brain. High expression levels of MANF in the CP and VZ/SVZ during embryonic development suggests that MANF may have multiple functions during cortical development.

### Lack of MANF does not affect NSC proliferation, self-renewal, or viability

**Figure 2.**
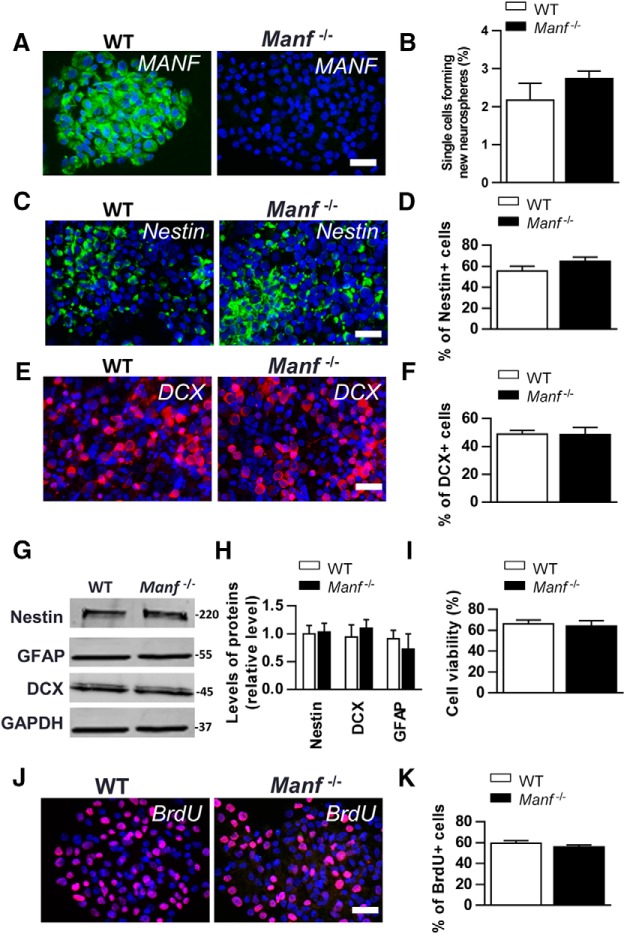
*In vitro* characterization of cultivated WT and *Manf^-/-^* NSCs. ***A***, Immunofluorescent staining with MANF (green) antibody shows high expression in WT neurospheres, and no signal in *Manf^-/-^* neurospheres. ***B***, There was no difference in the self-renewal assay in NSCs of *Manf^-/-^* and WT groups (*p* = 0.25, *n* = 4). Immunofluorescent staining with Nestin (green, ***C***), DCX (red, ***E***) antibodies and counterstained with DAPI (blue) in WT and *Manf^-/-^* NSCs. The percentage of Nestin-positive NSCs (***D***) and DCX-positive (***F***) neuronal precursor cells in WT and *Manf^-/-^* NSC cultures were analyzed by counting the ratio of Nestin- or DCX-positive cells to DAPI-positive nuclei, *n* = 3–5. NSCs were analyzed by Western blotting for Nestin, DCX, GFAP in WT and *Manf^-/-^* NSCs (***G***). Quantitative data from Western blottings (***H***) are presented as levels relative to GAPDH, *n* = 3–5. Numbers on the right show molecular weight as kDa. Cell viability did not differ between WT and *Manf^-/-^* NSCs (***I***). ***J***, ***K***, NSC proliferation assessed from BrdU-positive cells (red) as a ratio from DAPI-positive nuclei (blue) from NSC cultures, *n* = 5–6; scale bar: 50 µm.

Next, we studied the role of MANF in NSCs by establishing NSC cultures from E13.5 mouse brains. NSCs are multipotent and self-renewing to form neurospheres in culture. We found that most of the WT NSCs expressed MANF protein whereas *Manf^-/-^* NSCs were negative for MANF immunofluorescence (Fig. 2*A*). Using a self-renewal assay, where neurospheres are dissociated into single cells and their ability to form new neurospheres is measured, no difference was found between the two genotypes (Fig. 2*B*). Next, we studied whether MANF deletion affects the heterogeneous population of NSCs and stained WT and *Manf^-/-^* cells with the NSC marker Nestin, and the NPC marker DCX. There was no difference in the numbers of Nestin-positive or DCX-positive cells between the two genotypes (Fig. 2*C–F*). Also, Western blot analysis showed no difference between the genotypes of Nestin, GFAP, or DCX levels measured from neurospheres (Fig. 2*G*,*H*). Next, we measured whether MANF deletion affects the viability of NSCs and found that the number of trypan blue-stained dead cells was similar between the genotypes (Fig. 2*I*). The proliferation rate of cells, as expressed by the ratio of BrdU+ cells to the total cell number was also similar between the two groups (Fig. 2*J*,*K*). In short, MANF deficiency did not affect self-renewal capacity, viability, proliferation, or Nestin, DCX, and GFAP levels in cultured NSCs.

### Loss of MANF interferes with neuronal differentiation and impairs neurite outgrowth

**Figure 3.**
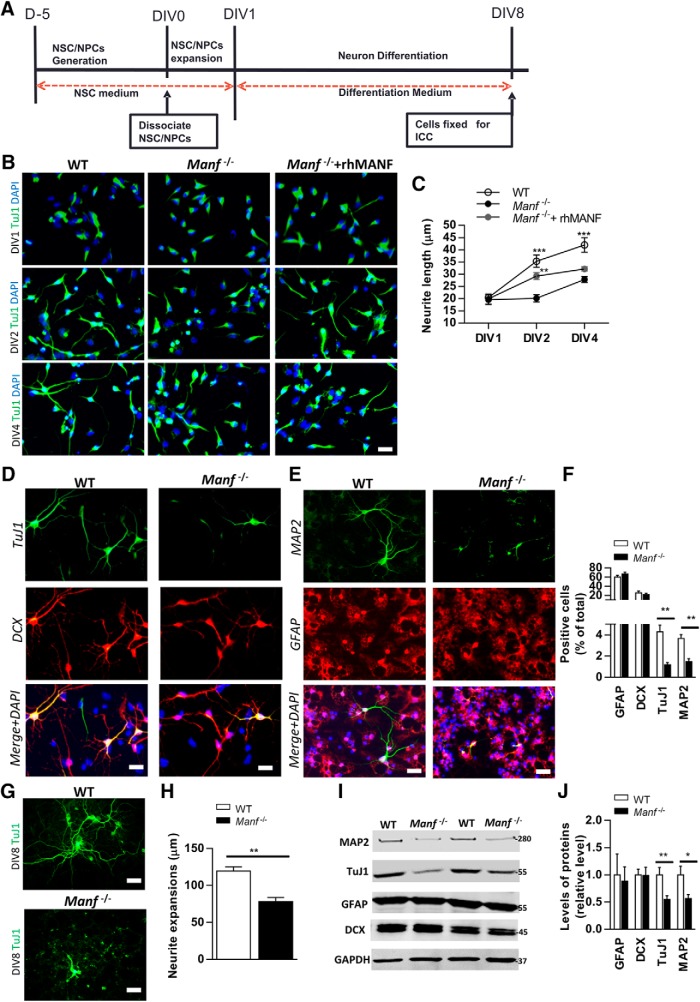
Effects of MANF on neuronal differentiation. ***A***, Time line of the differentiation of NSCs. NSC expansion was done for 1 d in floating conditions. At day 1, differentiation begun without EGF and FGF-2 for 7 d in adhering conditions. ***B***, At DIV1, DIV2, and DIV4, differentiated cells were stained with antibodies against neuronal markers TuJ1; scale bar; 50 µm. ***C***, Neurite length were measured from TuJ1-positive cells. *Manf^-/-^* cells displayed significantly shorter neurite length. Exogenous rhMANF induced neurite outgrowth at DIV2 but not at DIV4 of *Manf^-/-^* cells, two-way ANOVA, *post hoc* Bonferroni test; **p < 0.01, ***p < 0.001; scale bar: 50 µM. ***D***, ***E***, Representative photomicrographs of differentiated cells double-stained for TuJ1 (green), DCX (red), MAP2 (green), and GFAP (red), and nuclei stained with DAPI (blue); scale bar: 50 µM. ***F***, Ratios of GFAP-, DCX-, TuJ1-, and MAP2-positive cells in relation to DAPI+ nuclei (*n* = 9). ***G***, ***H***, At DIV8, neurite extension were measured from TuJ1-positive cells. *Manf^-/-^* cells displayed significantly shorter processes than WT cells (*n* = 9); scale bar: 20 µM. ***I***, Lysates from WT and *Manf^-/-^* cells after differentiation were immunoblotted with MAP2, TuJ1, GFAP, DCX, and GAPDH antibodies. ***J***, Protein levels were quantified in relation to levels of GAPDH, a housekeeping protein, *n* = 9; *p < 0.05, **p < 0.01, Student’s *t* test.

To investigate whether MANF is involved in neuronal differentiation and neurite growth *in vitro*, NSCs were allowed to differentiate in medium without mitogens (EGF, FGF-2) for 8 d (Fig. 3*A*). Initially, the changes in morphology of differentiated neurons from DIV1 to DIV4 (Video 1, WT; Video 2, *Manf^-/-^*) were studied and cells were stained with a TuJ1 antibody, a marker for postmitotic immature neurons (Fig. 3*B*). At DIV1, the WT and *Manf^-/-^* cells displayed slightly asymmetrical morphology. At DIV2, WT cells showed clear neurite outgrowth and increased neurite extensions at DIV4. Contrarily, *Manf^-/-^* cells did not show neurite outgrowth at DIV2, and at DIV4 neurite length was decreased compared to WT (Fig. 3*B*,*C*), indicating that loss of MANF disrupts neurite outgrowth. Therefore, we investigated whether decreased neurite outgrowth, caused by loss of MANF, can be improved by administration of rhMANF (200 ng/ml). Indeed, a single application of rhMANF could induce neurite outgrowth in *Manf^-/-^* cells at DIV2, but did not continue to promote the neurite extension at DIV4 (Fig. 3*B*,*C*), suggesting that extracellular MANF did not completely recover a deficit of neurite outgrowth from loss of endogenous MANF. At DIV8, we found that the number of DCX-positive cells was similar between the genotypes, but there were fewer TuJ1- and MAP2-positive cells in *Manf^-/-^* group compared to WT group (Fig. 3*D–F*, *p* < 0.01, Student’s *t* test). The number and morphology of GFAP-positive cells did not differ between the two groups (Fig. 3*E*,*F*), implying that loss of MANF only disrupts the neuronal differentiation *in vitro*. In addition, at DIV8 analysis of total neurite length revealed that Tuj1-positive *Manf^-/-^* cells displayed significantly shorter neurites compared to WT neurons (Fig. 3*G*,*H*). In line with the immunocytochemical staining, Western blotting revealed decreased levels of TuJ1 and MAP2, but not DCX or GFAP, in the *Manf^-/-^* group (Fig. 3*I*,*J*, *p* < 0.05, *p* < 0.01, respectively, Student’s *t* test). These results indicate that MANF plays a significant role in neuronal differentiation and neurite outgrowth.

**Video 1.** Video of WT cells.

**Video 2.** Video of MANF-/- cells.

### Altered thickness of the cerebral cortex in *Manf^-/-^* mice

**Figure 4.**
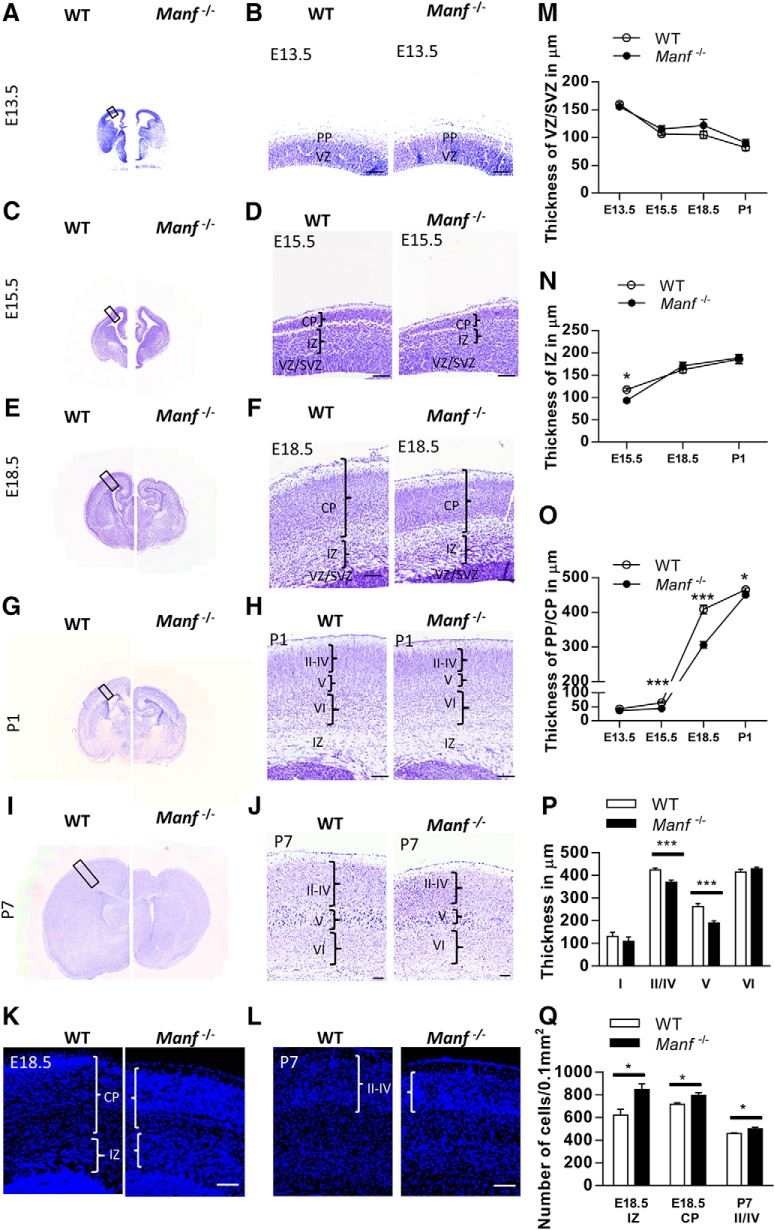
Morphologic alteration of embryonic and early postnatal cortex in WT and *Manf^-/-^* mice. ***A–J***, Coronal sections of the developing cortex stained with Nissl. ***A***, ***B***, There is no difference in the overall appearance of the cortex between WT and *Manf^-/-^* mice at E13.5. ***C***, ***D***, At E15.5, the IZ and CP are thinner in the *Manf^-/-^* mice than in WT littermates, but the thickness of SVZ/VZ is not changed. ***E***, ***F***, At E18.5, the CP is thinner in *Manf^-/-^* mice compared to WT mice. ***G–J***, At P1 and P7, the cortex layer is thinner, prominently at Layer II–V in *Manf^-/-^* mice compared to controls. ***K***, At E18.5, the cell density in the CP is higher in the *Manf^-/-^* mice compared to WT mice (blue, DAPI). ***L***, At P7, DAPI+ nuclei in the cortex Layer II–IV is higher in *Manf^-/-^* mice compared to WT mice. ***M***, There was no difference in VZ/SVZ thickness between WT and *Manf^-/-^* embryos or pups at any time point studied. ***N***, The thickness of IZ was significantly reduced at E15.5 in the *Manf^-/-^*, but recovered by E18.5 (***L***, *p < 0.05, Student’s *t* test). ***O***, The thickness of PP/CP was significantly reduced at E15.5, E18.5, and P1 in *Manf^-/-^* compared to WT littermates (***M***, ***p < 0.001, **p < 0.001, *p < 0.05, respectively, Student’s *t* test). ***P***, The thickness of cortical layers was measured at P7, and we observed significantly thinner Layers II/IV and V in *Manf^-/-^* mice compared to WT littermates (***p < 0.001, Student’s *t* test). ***Q***, Quantification of the DAPI+ nuclei; scale bars: 100 µm.

Given the observed effect of MANF knockout on neuronal differentiation *in vitro*, we investigated whether deletion of MANF affects the development of neocortex. We measured the thickness of WT and *Manf^-/-^* cortical structures after Nissl-staining during embryonic and postnatal corticogenesis. At E13.5, the histologic appearance of the cerebral wall in mutants and their WT littermates was similar (Fig. 4*A*,*B*). At E15.5, IZ and CP of the neocortex was thinner in *Manf^-/-^* embryos than in WT littermates (Fig. 4*C*,*D*,*N*,*O*). At E18.5, the CP was thinner in the *Manf^-/-^* group, and the thickness of IZ was similar between the genotypes (Fig. 4*E*,*F*,*N*,*O*). However, cell densities in the IZ and CP of the mutant brain were higher (Fig. 4*K*,*Q*). At P1 and P7, the thickness of the *Manf^-/-^* cerebral cortex was reduced, particularly the Layers II–IV and V, compared to the same area in WT cortex (Fig. 4*G–J*,*N*). Additionally, the corresponding higher cell density in Layer II–IV was observed in the mutant cortex at P7 (Fig. 4*L*,*Q*). In contrast, Layer VI was of normal size. The results indicate that loss of MANF *in vivo* does not affect the size of the VZ/SVZ but causes abnormal cerebral cortex development.

**Figure 5.**
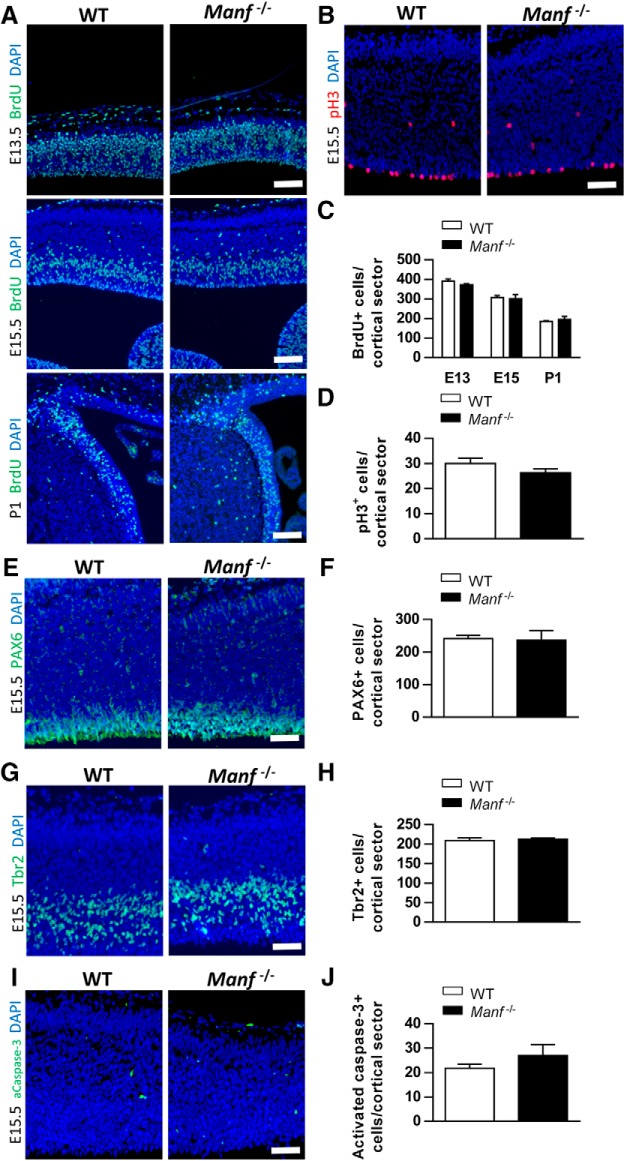
Loss of MANF does not affect the proliferation or survival of neural precursors. ***A***, ***C***, Analysis of S-phase progenitor cells by BrdU pulse labeling of WT and *Manf^-/-^* embryos at E13.5, E15.5, and P1. A similar number of S-phase cells are in the *Manf^-/-^* cortex relative to the WT littermates. The average number of BrdU-labeled cells in the SVZ was calculated at E13.5, E15.5, and P1. ***B***, ***D***, The number of pH3-positive cells in the VZ/SVZ of E15.5 *Manf^-/-^* mice is not different from WT littermates. The number of PAX6-positive cells (***E***, ***F***) or Tbr2-positive cells (***G***, ***H***) in the cortex did not differ between WT and *Manf^-/-^* brains. ***I***, ***J***, Very few apoptotic cells were found in cerebral cortex from E15.5 WT as well as *Manf^-/-^* mice immunostained with activated caspase-3. Cell nuclei were counterstained with DAPI (blue). Data are expressed as the mean ± SEM, *n* = 3-4; scale bars: 50 µm (***A***) and 100 µm (***B***, ***E***, ***G***, ***I***).

To determine if impaired *Manf^-/-^* neocortical development is caused by a decreased rate of proliferation in NSCs, we injected pregnant heterozygous *Manf^+/-^* mice or P1 pups with BrdU and harvested brain tissues 30 min later. BrdU immunofluorescence was performed on brain sections from E13.5, 15.5, and P1 animals to analyze cells in S-phase (DNA synthesis). pH3 immunofluorescence was conducted at E15.5 to analyze cells in M-phase (mitosis). The number of cells in S- or M-phase quantified from coronal sections was similar in the *Manf^-/-^* cortex compared to WT littermates (Fig. 5*A–D*). Additionally, quantification of the number of neuroepithelial cells, and intermediate progenitor cells, stained with PAX6 and TBR2, respectively, in the VZ/SVZ at E15.5, revealed no difference between the WT and *Manf^-/-^* group (Fig. 5*E–H*). To analyze programmed cell death, we performed immunohistochemistry for activated caspase-3. As depicted in Figure 5*I*,*J*, we found very few dying cells in sections from WT and *Manf^-/-^* neocortex. These data suggest that the deficits in the *Manf^-/-^* neocortical development is not due to the number of NSCs/NPCs, cell proliferation rate, or programmed cell death.

### The distribution of CTIP2+, Tbr1+, and Cux1+ cells is altered in *Manf^-/-^* neocortex

**Figure 6.**
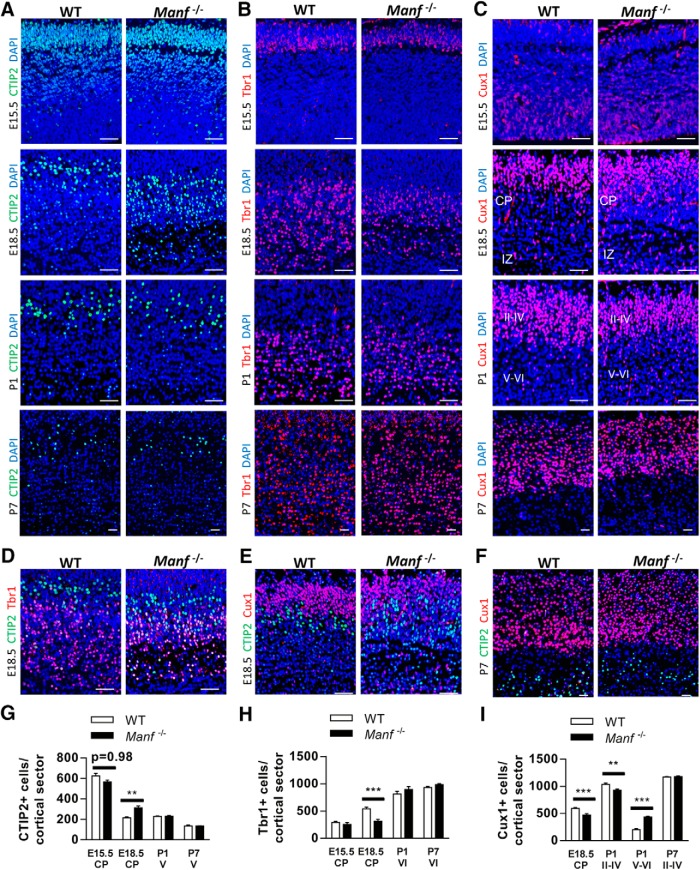
Distribution of cortical layer markers CTIP2, TBR1, and Cux1. ***A***, The number of CTIP2+ cells at E15.5 are similar between WT and *Manf^-/-^* in CP. At E18.5, there is a significant increase in CTIP2+ neurons density at deep layers of CP in the *Manf^-/-^* compared to the WT littermates, but there is no difference at P1 and P7. ***B***, Tbr1 is located at the lower CP in the WT controls and *Manf^-/-^* mice at E15.5. In the *Manf^-/-^* brain, there is a significant decrease in the number of Tbr1+ neurons in the deep layer of CP at E18.5 compared to WT controls, but there is a full recovery by P1 and P7. ***C***, At E15.5, Cux1 is located at the VZ/SVZ, but is absent in the CP in WT and *Manf^-/-^* brain. There are fewer Cux1+ neurons in the superficial CP at E18.5 and Layers II–IV at P1 of *Manf^-/-^* brain than in WT controls. In the *Manf^-/-^* mouse brain, more Cux1-positive neurons remained in the IZ at E18.5 and in the Layers V and VI at P1. At P7, there is no difference in Cux1-positive neurons in the cortex between the genotypes. ***D***, Representative photomicrographs of E18.5 CP stained for Tbr1 (red), CTIP2 (green), and nuclei (DAPI, blue). ***E***, Representative photomicrographs of E18.5 CP stained for Cux1 (red), CTIP2 (green), and nuclei (DAPI, blue). ***D***, Representative photomicrographs of P7 cortex stained for Tbr1 (red), CTIP2 (green), and nuclei (DAPI, blue). ***G***, The number of CTIP2+ cells in the CP at E15.5 and E18.5 and Layer V at P1 and P7 (*n* = 3; **p < 0.01). ***H***, The number of Tbr1+ neurons in the CP at E15.5 and E18.5 and at Layer VI at P1 and P7 (*n* = 3; ***p < 0.001). ***I***, The number of Cux1+ neurons in the CP at E18.5; Layers II–VI, V–VI at P1 and P7 (*n* = 3; **p < 0.01, ***p < 0.001); scale bar: 100 µm.

To study how MANF deficiency influences cortex development, cortical sections from WT and *Manf^-/-^* mice were stained with CTIP2, Tbr1, and Cux1: markers for neuronal specification in different layers during cortex development (Molyneaux et al., 2007; Bedogni et al., 2010). As expected, at E15.5, CTIP2+ cells were found in the whole CP while Tbr1+ cells were mainly found in the lower part in both genotypes (Fig. 6*A*,*B*). Moreover, the numbers of CTIP2+ and Tbr1+ neurons were similar in WT and *Manf^-/-^* embryos (Fig. 6*G*,*H*). At E18.5, considerable Tbr1+ cortical neurons accompanied by scanty CTIP2+ neurons have been found at the lower layer of the cortex in WT embryos. In contrast, in *Manf^-/-^* embryos, there were fewer Tbr1+ neurons, but more CTIP2+ subcerebral projection neurons at the lower layer of the cortex (Fig. 6*A*,*B*,*G*,*H*). Moreover, the distributions of CTIP2+ and Tbr1+ neurons are partially intermingled in the mutant CP (Fig. 6*D*), unlike in WT embryos, having them being split into different layers, implying that many subcerebral neurons in *Manf^-/-^* embryos did not migrate to their destined positions or did not yet acquire layer-specific identification. At P1 and P7, the total number of CTIP2+ and Tbr1+ neurons in Layers V/VI were similar between the two genotypes.

We next analyzed Cux1+ neurons in developing cortex. Cux1+ cells are found in SVZ from E14.5 onwards, and during postnatal days, Cux1+ cells migrate to Layers II–IV (Cubelos et al., 2010). At E15.5, there were few Cux1+ neurons in the CP in both WT and *Manf^-/-^* embryos (Fig. 6*C*). At E18.5, many of the Cux1+ neurons were found in the upper layer of CP in WT mice. However, in *Manf^-/-^* mice, there were fewer Cux1+ neurons in the CP compared to WT littermates (Fig. 6*C*,*I*). Also, different to the WT group, with a clear distribution of subtype neurons, in mutant embryos Cux1+ and CTIP+ neurons were dispersed in the CP, revealing a disordered neuronal arrangement (Fig. 6*E*). At P1, more Cux1+ neurons were found in the deeper layers in *Manf^-/-^* mice than in WT littermates, implying that *Manf^-/-^* neurons had a delay in radial migration (Fig. 6*C*,*I*). At P7, the total number of Cux1+ neurons was similar between the genotypes and the arrangement of *Manf^-/-^* cortical neurons was fully recovered due to higher cell density observed at the Layers II–IV owing to a thinner cortex in mutant mice. Taken together, these findings suggest that the thinner CP accompanied by the abnormal distribution of cortical layer markers in *Manf^-/-^* embryos might be caused by slower neuronal migration and disturbed subtype neuron specification.

### Delayed neuronal migration in MANF-deficient embryos

**Figure 7.**
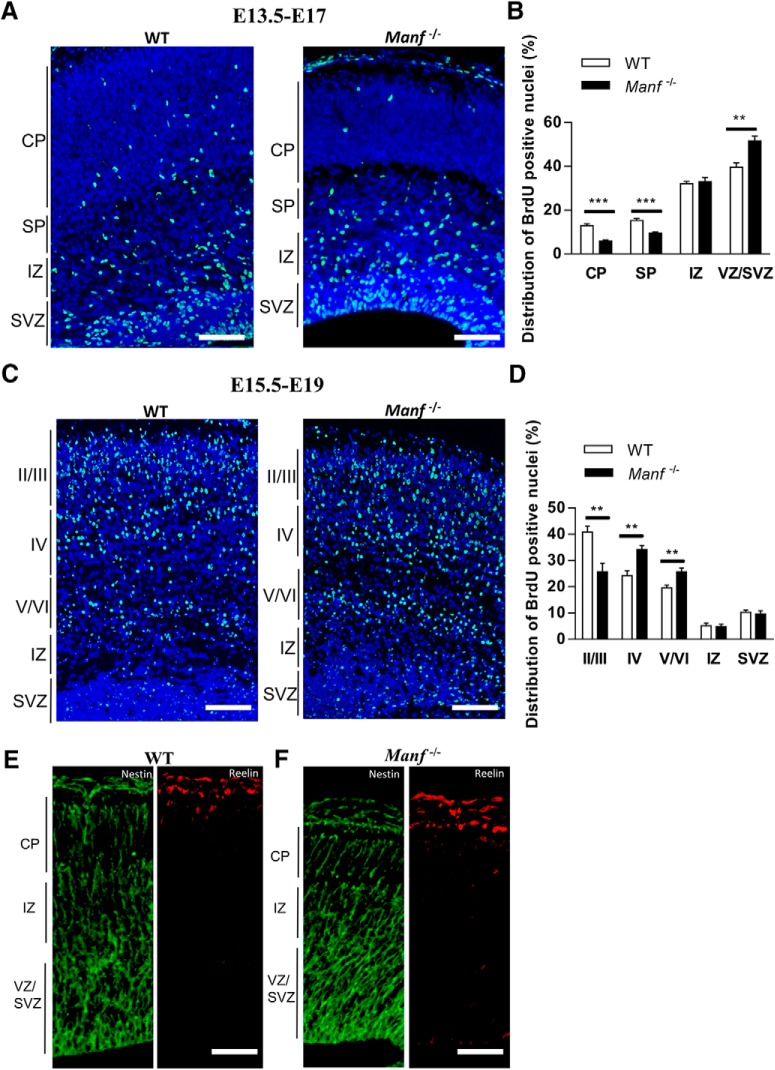
Radial migration of early (E13.5) and later (E15.5) born neurons in the neocortical regions with BrdU birth dating. ***A***, BrdU immunofluorescence staining of E17 cortex (BrdU administrated to pregnant *Manf^+/-^* mice at E13.5). ***B***, The number of BrdU-labeled cells in each layer was counted and shown as percentages of total labeled cells number in the neocortex. The relative number of BrdU-positive cells were found to be decreased in CP and SP of *Manf*^-/-^ mice compared to WT littermates, and increased levels in VZ/SVZ (*n* = 3; **p < 0.01, ***p< 0.001). ***C***, BrdU was administered at E15.5 and the distribution of the labeled cells was examined at E19. In the WT cortex, increased number of labeled cells (green) were found in in the Layers II/III compared to that in *Manf^-/-^* cortex. In contrast, increased number of BrdU-positive cells were found in the cortical Layers IV and V/VI in the *Manf^-/-^* mice compared to the WT mice. ***D***, To quantify the distribution of BrdU-positive nuclei, the cerebral cortex was divided into five area from the pial side to the ventricular side. The number of labeled cells in each bin was counted and shown as percentages of total labeled cells number in the CP (*n* = 3, *p < 0.05, ***p < 0.001 for *Manf^-/-^* mice compared to WT). Nuclei were counterstained with DAPI (blue). ***E***, ***F***, Immunofluorescent staining of coronal sections of neocortex with anti-Nestin and anti-Reelin antibodies. Radial glial architecture and Reeling expression in were similar in WT and in mutants; scale bars: 50 µm (***A***, ***C***) and 25 µm (***E***, ***F***).

To examine whether MANF regulates neuronal migration in the cerebral cortex, we used BrdU birth dating. We first labeled the early-generated neurons by injecting pregnant *Manf^+/-^* mice with BrdU at E13.5 and analyzed the embryos at E17 (Fig. 7*A*). Cortical sections from E17 embryos were stained with BrdU antibodies, and the number of BrdU+ cells were quantified in each cortical layer. The number of BrdU+ cells detected in the CP and SP of *Manf^-/-^* embryos were significantly reduced compared to WT littermates (Fig. 7*A*,*B*). In contrast, the number of BrdU+ cells in VZ/SVZ of *Manf^-/-^* embryos were significantly increased compared to WT. Next, we labeled later generated neurons by administering BrdU to pregnant *Manf^+/-^* females at E15.5 and BrdU-labeled nuclei were examined in the CP at E19 (Fig. 7*C*,*D*). Most BrdU-labeled nuclei were located at Layer II/III of WT cortex. However, in *Manf^-/-^* cortex, more BrdU-labeled nuclei were found in Layers IV and V/VI (Fig. 7*C*,*D*). Thus, these two experiments indicate that radial migration of both early- and later-generated neurons is impaired in *Manf^-/-^* embryos. Moreover, we examined the overall architecture of radial glial fibers by Nestin staining and the expression patterns of Reelin, a matrix glycoprotein that regulates neuronal migration and positioning. We found that Nestin and Reelin immunofluorescence are similar in WT and *Manf^-/-^* cortices at E15.5 (Fig. 7*E*,*F*) suggesting that the deficits in neuronal migration and positioning are characteristic of immature *Manf^-/-^* neurons. Overall, these results suggest that MANF is essential for migration of neurons in the embryonic cortex.

### Decreased neurite extension and abnormal neuronal architecture in the *Manf^-/-^* cortex

**Figure 8.**
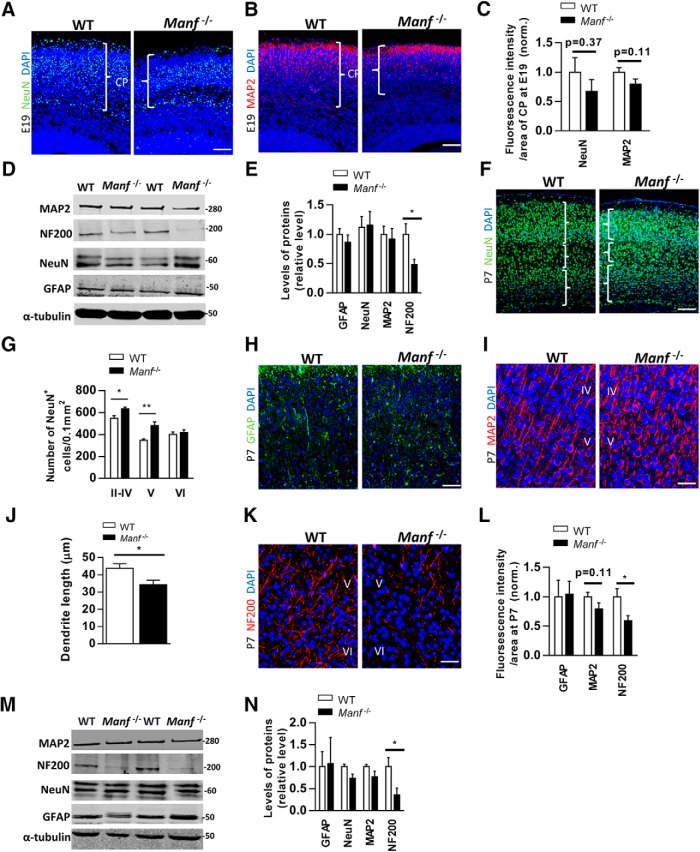
NeuN, GFAP, MAP2, and NF200 expression in the developing cortex. ***A***, ***B***, Representative photomicrographs of WT and *Manf^-/-^* cortical sections stained with NeuN (***A***) or MAP2 (***B***) at E19, and counterstained with DAPI (blue) at E19. ***C***, The fluorescent intensities divided by the area of CP. There is no difference between WT and *Manf^-/-^* embryos in the NeuN and MAP2 fluorescent intensities/area of CP (*n* = 3). ***D***, ***E***, Western blotting from E19 cortical lysates stained with antibodies against NeuN, MAP2, GFAP, and NF200 (*n* = 4, *p < 0.05). ***F***, Representative photomicrographs of anti-NeuN (green) antibody-stained cortical sections of WT and *Manf^-/-^* mice at P7, counterstained with DAPI (blue). ***G***, The density of NeuN-positive neurons in cortical layers at P7. In the *Manf^-/-^* mouse brain there is a significant increase in NeuN-positive neuronal density in the Layers II–IV and V at P7 (*n* = 3, *p < 0.05, **p < 0.01). ***H***, Representative photomicrographs of WT and *Manf^-/-^* cortical sections stained with GFAP. ***I***, Representative photomicrographs of WT and *Manf^-/-^* cortical sections stained with MAP2. ***J***, Shorter MAP2-stained dendrites were found in *Manf^-/-^* cortical neurons at Layer IV/V of cortex compared to those in WT (*n* = 3, *p < 0.05). ***K***, Layer V/VI of cortex from *Manf^-/-^* and WT littermates stained with neurofilament antibody (NF200). Cell nuclei were counterstained with DAPI. ***L***, At P7, there is no difference in immunofluorescence intensities of MAP2 and GFAP-stained cortical sections between groups. However, there was a significant decrease in axon-specific neurofilament immunoreactivity in *Manf^-/-^* cortex (*n* = 3, *p < 0.05). ***M***, ***N***, Western blotting from P7 cortical lysates probed with glial and neuron-specific antibodies. Decreased levels of NF200 was detected in the cortical extracts from *Manf^-/-^* mice (*n* = 4, *p < 0.05); scale bars: 50 µm (***A***, ***B***, ***F***, ***H***) and 25 µm (***I***, ***K***).

Considering that loss of MANF caused reduced thickness of the cortical layer, we investigated whether the lack of MANF decreases the number of neurons or glial cells at E19 and P7. At E19 when cortical lamination is being developed, few NeuN-positive neurons and MAP2-positive dendrites were located in the CP (Fig. 8*A*,*B*). The fluorescent signal intensities of NeuN and MAP2 in relation to the area of CP showed no significant differences between the two genotypes (Fig. 8*C*), suggesting that loss of MANF does not affect the generation of cortical neurons. Similarly, Western blot analysis showed that NeuN and MAP2 levels from E19 CP tissues were similar between the genotypes (Fig. 8*D*,*E*). Additionally, GFAP levels were similar between genotypes, but surprisingly, the level of neurofilament (NF200) was significantly lower in *Manf^-/-^* CP. Since neurofilament exerts a regulatory function in cell structure, axonal transport and stabilization (Lariviere and Julien, 2004), these data might support the role of MANF in axon outgrowth during later cortical development. At P7 we found no difference in the number of NeuN+ neurons between the two genotypes (Fig. 8*F*). However, when investigating the neuronal distribution in the cortex, we found increased NeuN density in the Layers II–V, but not Layer VI, of the *Manf^-/-^* cortex (Fig. 8*F*,*G*). Additionally, the fluorescent intensity of GFAP was similar in the two genotypes (Fig. 8*H*). While there was no difference in MAP2 fluorescent intensity between the genotypes (Fig. 8*I*,*J*), *Manf^-/-^* neurons displayed shorter MAP2-stained dendrite extensions compared to WT littermates (Fig. 8*J*). Further, axons from *Manf^-/-^* cortex stained less intensely with the neurofilament marker compared to WT axons (Fig. 8*K*,*L*). In line with the above results, the levels of GFAP, MAP2, and NeuN in Western blotting were similar between the two genotypes (Fig. 8*M*,*N*). The NF200 level was reduced in *Manf^-/-^* cortex compared to WT at P7 (Fig. *8M*,*N*). Overall, these findings suggest that loss of MANF does not decrease the number of cortical neurons or glial cells, but results in decreased neurite extension as well as axon-specific cytoskeletal protein levels, and abnormal neuronal density in the developing cortex.

### Loss of MANF results in activated UPR but not increased apoptosis during NSC differentiation

**Figure 9.**
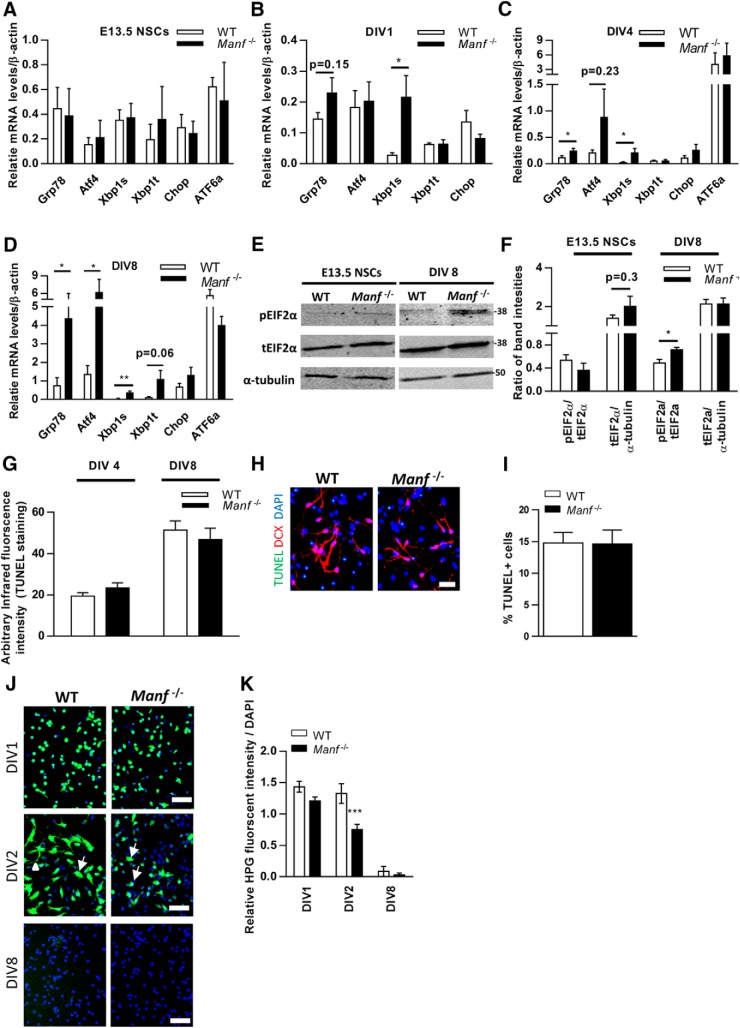
Unfolded protein-response genes are upregulated, and increased levels of p-eIF2α protein is found in the *Manf^-/-^* NSCs during differentiation. ***A-D***, qPCR analysis of UPR genes *Atf4*, *Grp78*, *Chop*, *Xbp1s*, *Xbp1t* in WT and *Manf^-/-^* NSCs (***A***), and differentiated cells at DIV1 (***B***), DIV4 (***C***), and DIV 8 (***D***), *n* = 8-11, *p < 0.05, **p < 0.01, Student’s *t* test. ***E***, Western blotting from NSCs and differentiated cells at DIV8. ***F***, Quantified intensities of Western blotting bands of p-eIF2α was compared to total amount of (t)eIF2α and teIF2α to intensities of α-tubulin, *n* = 4, *p < 0.05, Student’s *t* test. ***G*,** TUNEL infrared fluorescence intensities were similar from cultures obtained from *Manf^-/-^* and WT differentiated cells at DIV4 and DIV8. ***H*,** Representative photomicrographs of differentiated cells stained for TUNEL (green), DCX (red), and nuclei were counterstained with DAPI (blue); scale bar: 50 µM. ***I***, Differentiated NSCs at DIV8 death assessed by TUNEL staining. ***J*,** WT and *Manf^-/-^* NSCs at DIV1, DIV2, and DIV8 incubated in HPG. Diagonal arrows denote the cytoplasmic localization of synthesized proteins and arrowhead indicates newly synthesized proteins spread to growing neurites. ***K***, Quantified ratio of Click-iT HPG fluorescence to DAPI fluorescence intensities in WT and *Manf^-/-^* cells, two-way ANOVA, *p* < 0.01, Bonferroni’s *post hoc* test, ***p < 0.001.

Previous studies have indicated that MANF is upregulated in ER stress conditions *in vitro* (Apostolou et al., 2008; Glembotski et al., 2012) and lack of MANF leads to chronic UPR activation in pancreatic islets *in vivo* (Lindahl et al., 2014). It has also been shown that dysregulation of UPR can cause defects in neurogenesis and neuronal maturation and that these mechanisms could underlie neurodevelopmental disorders (Godin et al., 2016). Therefore, we wanted to investigate whether increased ER stress and activated UPR also appear in *Manf^-/-^* NSCs and measured the expression levels of UPR genes Grp78, Atf4, Atf6a, spliced Xbp1, and Chop, which are known to increase under ER stress conditions. In undifferentiated NSCs there was no difference in the levels of mRNAs for *Chop*, spliced *Xbp1*, total *Xbp1, Atf4*, *Atf6a*, and *Grp78* (alias *Bip*) between the *Manf^-/-^* group and WT controls (Fig. 9*A*). However, at DIV1, the level of spliced *Xbp1* mRNA expression was higher in the *Manf^-/-^* group, followed by increased Grp78 at DIV4 (*p* < 0.05, Student’s *t* test; Fig. 9*B*,*C*). At DIV8, significant elevation of mRNA levels for *Grp78*, *Atf4*, and spliced *Xbp1* were found in *Manf^-/-^* differentiated cells (Fig. 9*D*). Moreover, quantification of p-eIF2α band intensities compared to total levels of (t)eIF2α by Western blot analysis revealed a higher level of p-eIF2α compared to total EIF2α in *Manf^-/-^* NSCs at DIV8 (Fig. 9*E*,*F*). Collectively, these studies show that when *Manf^-/-^* NSCs start to differentiate, Grp78 and spliced *Xbp1* mRNA in the inositol-requiring enzyme 1 (IRE1) pathway are upregulated, followed by increased phosphorylation of eIF2α protein and *Atf4* mRNA demonstrating the activation of the protein kinase RNA-like ER kinase (PERK) pathway. Meanwhile, *Atf6a* or *Chop* mRNA levels were not altered during *Manf^-/-^* neuronal differentiation.

Next, by using TUNEL staining In-Cell Westerns assay and immunofluorescent analysis we compared the apoptotic rate of differentiated cells between WT and *Manf^-/-^* group. With In-Cell Westerns assay, we observed similar levels of TUNEL infrared fluorescent intensity in WT and *Manf^-/-^* NSCs at DIV4 or DIV8 (Fig. 9*G*). Consequently, quantification of TUNEL+ cells revealed equal levels of apoptotic cells in WT and *Manf^-/-^* NSCs at DIV8 (Fig. 9*H*,*I*). Thus, we suggest that loss of MANF results in increased UPR activation, which does not lead to cell death but rather might underlie the defects in neuronal differentiation seen in *Manf^-/-^* NSCs.

### Loss of MANF decreases *de novo* protein synthesis at the beginning of NSC differentiation

The experiments described above indicate that loss of MANF increases ER stress and UPR signaling, and thus disturbs ER homeostasis during neuronal differentiation. Since activation of PERK and IRE-1 pathway directly regulate proteins synthesis, we wanted to study whether MANF deficiency further disturbs protein synthesis or processing when NSCs differentiate. We implemented a HPG-based assay for the detection of nascent protein synthesis. At DIV1, most WT and *Manf^-/-^* NSCs showed strong HPG-derived signals (Fig. 9*J*). After differentiation, at DIV2, most WT cells showed intense fluorescence in the cell soma and neurites (Fig. 9*J*). These signals are likely to reflect the presence of newly synthesized proteins localized in the neuronal soma (arrows) and disseminated to growing neurites (arrowhead). However, *Manf^-/-^* cells had less HPG-derived signal compared to WT. Also, the signals in *Manf^-/-^* cells were limited to soma, which could explain the neurite sprouting defects in *Manf^-/-^* cells have not yet developed neurite sprouting. Quantification of the signal intensities revealed significantly lower intensity in differentiating *Manf^-/-^* NSCs (DIV2; Fig. 9*K*). These results suggest that MANF deficiency results in a reduction of newly synthesized proteins at the beginning of cell differentiation. When we analyzed the newly synthesized proteins at DIV8, the very weak fluorescence signal was found both in WT and *Manf^-/-^* cells (Fig. 9*K*), indicating that after differentiation there are less newly synthesized proteins needed.

## Discussion

Previous studies have shown that exogenously added MANF has neuroprotective and neurorestorative properties (Airavaara et al., 2009; Voutilainen et al., 2009; Yang et al., 2014; Neves et al., 2016), and *in vitro* ER localization is needed for neuroprotection (Hellman et al., 2011; Mätlik et al., 2015). In our study using MANF antibody validated on MANF knockout tissues, we found that MANF is not only expressed in mature postmitotic neurons but that it is also expressed at high levels in the mitotic NSCs. We demonstrate for the first time that MANF is highly expressed in the neurogenic areas in the mammalian brain. Therefore, we focused on studying its role in NSCs *in vitro* and *in vivo* using MANF-deficient neurosphere cultures and developing mouse cortex, respectively.

By isolating NSCs from *Manf^-/-^* embryos, we discovered that when cultured *in vitro*, MANF-deficient NSCs have impaired neurite growth, and this was further verified in developing mammalian cortex *in vivo.* These findings raise the question of why the loss of MANF influences neurite growth but does not compromise cell proliferation or cell survival. Recent studies have indicated that MANF is an ER luminal protein, and interacts with the chaperone GRP78, and is secreted in response to ER stress *in vitro* (Mizobuchi et al., 2007; Apostolou et al., 2008; Glembotski et al., 2012; Mätlik et al., 2015; Lindahl et al., 2017). Three branches of UPR that are initiated by distinct ER stress transducers located on the ER membrane: PERK, IRE1, and activating transcription factor 6 (ATF6). Under basal conditions, these proteins are bound by the ER chaperone Grp78 (also known as Bip) and maintained in an inactive state (Bertolotti et al., 2000). When ER stress develops, GRP78 is sequestered by the misfolded polypeptides and, consequently, released from the three sensor proteins, which triggers activation of the UPR branches (Hetz, 2012). UPR restores protein homeostasis by suppressing protein translation, inducing ER-related molecular chaperones to promote refolding of unfolded proteins, removing unfolded proteins by activating the ER-associated protein degradation system and promoting cell survival. If the UPR fails to restore protein homeostasis in the cell, prolonged and unmitigated ER stress leads to CHOP-mediated cell death. During neural differentiation, dendrites acquire their morphology by branch sprouting, which is accompanied by an increased need for protein synthesis (Ramírez and Couve, 2011). Increased protein synthesis can trigger UPR, a homeostatic signaling pathway to cope with the increased demand for protein folding (Torre and Steward, 1996; Ju et al., 2004; Murakami et al., 2007; Ramírez and Couve, 2011). However, prolonged ER stress when UPR fails to restore the normal function of the ER causes aberrant neuronal differentiation, retardation in neurite outgrowth, or even cell death (Kitao et al., 2004; Galehdar et al., 2010; Lin et al., 2012; Zhao et al., 2013; Kawada et al., 2014). While the downstream targets of the UPR pathway are not well studied in differentiating neurons, increased eIF2α phosphorylation is known to lead to a global decrease in mRNA translation initiation and ATF4 upregulation, which disrupts cAMP–induced responses and interferes with neuronal differentiation (Roffé et al., 2013). *In vitro*, rhMANF treatment could restore neurite outgrowth at the beginning of differentiation, although it did not improve neurite extension in *Manf^-/-^* cells at later time points. It should be noted that rhMANF was administered only once, and multiple administrations could have caused a more robust restorative effect. Since in postmitotic neurons MANF *in vitro* has not been shown to bind to cell surface receptors or protect cells (Hellman et al., 2011), our results suggest that MANF has different effects on undifferentiated cells. Whether the effect is mediated by MANF functioning specifically in the ER remains unknown. Nevertheless, this finding implies that extracellular MANF modulates the UPR to rescue neurite growth partially when mutant cells start to differentiate. Moreover, following increased need for protein synthesis and processing, *Manf^-/-^* cells seem to have difficulties in coping with the increased load of unfolded proteins, which further increases ER stress and subsequently impairs neuronal differentiation leading to shorter neurite extensions. The levels of CHOP and ATF6 did not increase significantly during neuronal differentiation and lack of increase can explain why the loss of MANF does not compromise cell survival. Considering that ∼30% of newly synthesized proteins are misfolded during differentiation (Schwarz et al., 2004), it is likely that lack of MANF, interfering with ER homeostasis, causes deficits in the protein processing, transport, and secretion needed for the morphologic changes during neurite growth.

*Drosophila melanogaster* embryos lacking both maternal and zygotic DmMANF show there are robust deficits in axonal arborization of dopamine neurons (Palgi et al., 2009). However, it is important to note that DmMANF is dominantly expressed in glial cells surrounding dopaminergic neurons, which suggests that the neuronal maturation-promoting factor in the fly originates from glial cells. In addition, knockdown of MANF expression with antisense splice-blocking morpholino oligonucleotides in *Danio rerio* revealed that MANF is necessary for the development of the dopaminergic neurons (Chen et al., 2012). Surprisingly, deletion of MANF in mice leads to a progressive postnatal reduction of pancreatic β-cells followed by insulin-deficient diabetes (Lindahl et al., 2014). Different from invertebrate species (Palgi et al., 2009), MANF protein in the mammalian brain is widely localized in neural lineage cells, suggesting that MANF has important functions in neuronal development. During neuronal differentiation, neurite growth is a crucial step and regulated by multiple signaling pathways. In the developing cortex, deletion of MANF disturbs neurite growth. However, there is no decrease in the number of neurons in the mutant cortex, which was observed in the *in vitro* experiments. This discrepancy may be the result of the distinctive environments between *in vitro* and *in vivo*. Disruption of the three-dimensional environment, cell-cell contacts as well as the extracellular environment are limitations in *in vitro* experiments (Gordon et al., 2013). Therefore, cultivated *Manf^-/-^* NSCs, which endure with increasing ER stress in a non-physiologic environment have difficulties to differentiate into neurons whereas, *in vivo*, extracellular environment and cell-cell connections can support *Manf^-/-^* neural precursor cells during differentiation. Otherwise, our data are difficult to interpret regarding the recent finding of MANF modulating immune system (Neves et al., 2016). Taken together, our results demonstrate a role of MANF in regulating the neurite extensions during the development of cerebral cortex.

In the developing cortex, the migration of newborn cells is directed away from the VZ/SVZ and radial migration is the main mechanism that creates the layer structure of excitatory neurons in the neocortex. In this study, a *Manf^-/-^* at E18.5 had at thinner CP layer likely because early- and late-born neurons had not yet arrived to their destined positions. Following neuron migration to their specific layers of the cortex, the deficit is partially recovered at postnatal stages. Additionally, based on our neuronal birth-dating analysis, *Manf^-/-^* mouse cortical neurons did not exhibit an inverted (outside-in) laminar formation pattern. This difference in migratory defects suggests that the mechanism in *Manf^-/-^* mice is distinct from that seen in reeler and related mutant mice (Mimura et al., 2008). Another explanation is that truncated or reduced organization of radial glial cells is responsible for the perturbed neuronal migration (Noctor et al., 2004). However, in *Manf^-/-^* mice, the radial glial scaffold, which is stained by Nestin, was not altered. Actually, we found that loss of MANF causes shorter neurite extensions and lower levels of NF200. It has been shown that when neural precursor cells start to leave from the VZ/SVZ, they develop leading and trailing processes to exert cell migration (Hirai et al., 2006). Also, axon-like processes would continue to grow during radial migration of neocortical pyramidal neurons (Hatanaka and Murakami, 2002). Several studies have demonstrated that developing axons undergo sequential neurofilament-mediated stabilization in a proximal-distal manner, which supports continued axonal outgrowth (Lariviere and Julien, 2004; Yuan et al., 2012; Lee and Shea, 2014). Accordingly, neuronal migration and neurite growth are correlated and can depend on the same molecular mechanisms (Yamasaki et al., 2011). Given the importance of MANF in neurite growth, it is not surprising that MANF plays a significant role in the regulation of neuronal migration.

Nonetheless, loss of MANF, disturbing the process of neurite extensions and maturation, may also interfere with the time course of subtype generation of subcerebral projection neurons and lead to the compacted architecture with increased neuronal density at the postnatal stage. In mouse embryos, expression of CTIP2, a zinc finger transcription factor, is observed in young neurons as early as E11.5, when subcerebral neurons are generated (Leone et al., 2008). Then when deeper layer neurons acquire the specification of distinct subtypes, SP neurons, and Layer VI neurons (corticothalamic neurons) are characterized by high levels of Tbr1 expression, but low CTIP2 expression (Morimoto-Suzki et al., 2014). Otherwise, the Layer V neurons are represented by the CTIP2 marker. At a later point in the embryogenic stage, when upper layer neurons are generated, Cux1 is mostly expressed in corticocortical projection neurons of Layer II–IV (Cubelos et al., 2010). Our results indicate that the subtype specification is disrupted in *Manf^-/-^* mice, but the reason for this remains unknown. Further studies will need be done to investigate whether MANF affects transcription factors regulating the production of neuronal subtypes in a spatiotemporal manner (Alcamo et al., 2008; Chen et al., 2008; Bedogni et al., 2010), whether MANF directly controls the generation of Layer V or VI neurons as well as their distribution during development.

We have previously shown that *Manf^-/-^* mice suffer from a severe growth defect (Lindahl et al., 2014). However, with respect to brain and body weight ratio, we found no differences in the brain/body weight (%) between genotypes at P56 (males: WT 1.9 ± 0.0, *n* = 4; *Manf^-/-^* 2.4 ± 0.2, *n* = 4 and females: WT 2.5 ± 0.1, *n* = 7; *Manf^-/-^* 2.8 ± 0.2, *n* = 7). Notably, conventional MANF-KO adult mice develop diabetes (Lindahl et al., 2014). At P28 serum insulin levels are already greatly decreased in mutant mice. Despite no change in random fed blood glucose levels in *Manf^-/-^* mice at P14, decreased glucose tolerance was found (Lindahl et al., 2014). As high blood glucose levels are implicated in affecting the development of different parts of the brain (Tayman et al., 2014), we used brains from embryonic and early postnatal *Manf^-/-^* mice to investigate the time-dependent differences in the cortical layer thickness. Thus, the thinner cortical layers in mutant embryos and pups are attributed to delayed neuronal migration and aberrant neuronal density. Brain-specific Nestin-Cre±::Manf^fl/fl^ mice are viable and do not show any gross defects (Lindahl et al., 2014; Pakarinen and Lindahl, unpublished results). However, as MANF is still expressed in some cells in the brain of conditional Nestin-Cre±::Manf^fl/fl^ brains (Pakarinen and Lindahl, unpublished results), we did not find this model suitable for our study.

Our study shows that MANF is not only expressed in postmitotic neurons but also in mitotic NSCs in the mammalian brain. We demonstrate that MANF does not affect self-renewal capacity or proliferation of NSCs and suggest that MANF is dispensable for neurogenesis. However, MANF plays a significant role in neurite growth in the process of neuronal differentiation and this, in turn, is likely the cause for delayed neuronal migration. This study is the first to demonstrate that MANF regulates neuronal extension in mammalian neurons and there is decreased in *de novo* protein synthesis and chronic ER stress on differentiation. In conclusion, these results suggest that MANF has a fundamental role in ER homeostasis during neurite growth and neuronal migration.

## Synthesis

Reviewing Editor: Chay Kuo, Duke University Decisions are customarily a result of the Reviewing Editor and the peer reviewers coming together and discussing their recommendations until a consensus is reached. When revisions are invited, a fact-based synthesis statement explaining their decision and outlining what is needed to prepare a revision will be listed below. The following reviewer(s) agreed to reveal their identity: Stephanie Gupton, Songhai Shi.

Reviewer #1

This paper examines the role of Mesencephalic astrocyte-derived neurotrophic factor (MANF) in neuronal differentiation and development. Interestingly although a member of a neurotrophic factor family, this study demonstrates that loss of Manf has no effect on proliferation or survival of neuronal stem cells, both in vitro and in vivo. Rather loss of Manf affects neuronal differentiation and development in vitro, and neuronal development and migration in vivo. They also show that loss of Manf is associated with ER stress, UPR, and a reduction of protein synthesis. These defects likely underlie these inhibitory effects on neurite outgrowth and potentially migration. This study is clearly written, with beautiful and well controlled data. I recommend publication with only the minor editorial revisions to the manuscript.

1. The Comments on why neurodifferentiation was impaired in vitro but not in vivo in the absence of MANF were appreciated. Could you also add a sentence or two discussing why addition of extracellular MANF partially rescued neuronal development, if it is indeed functioning as an ER resident protein.

2. Clarify that chop, xpba, atf4, atf6a and grp78 are markers of UPR.

3. Clarify if the Manf-/- mice are germ line deletion/complete null.

4. Reduce the numbers on figure 9D y axis

5. What type of posthoc corrections were performed on ANOVA?

6. Were all data normally distributed?

Reviewer #2

In this manuscript, Tseng et al. investigated the function of mesencephalic astrocyte-derived neurotrophic factor (MANF) in the developing mouse neocortex. Taking advantage of the Manf knockout mice, the authors performed a series of in vivo and in vitro analyses, and concluded that MANF is crucial for neuronal differentiation, neurite growth, and neuronal migration likely by regulating ER homeostasis and unfolded protein response (UPR).

This is a solid study and provides important information on the function of MANF in regulating mammalian neocortical development.

Main Comments:

1. The authors presented substantial in vitro and in vivo experimental data. Notably, there are some differences/discrepancies between in vitro and in vivo data. As the authors suggested, this might be due to different experimental contexts. Given that the knockout mice are available and viable, it may be good to focus on the in vivo data. It will also be easier for the readers, as it is somewhat hard to follow two lines of experiments with inconsistent results/interpretations.

2. The quality of some images in the figures can be improved. For example, in Figure 6, it would be good to show the images of the entire cortex to clearly illustrate the migration defects. Also, the Golgi staining images in Figure 8M are of poor quality, as the stained cells appear to be mostly glia, but not neurons. High magnification images of representative neurons should be provided.

3. The authors should extend the phenotype analysis to a more mature time point, e.g. P30, as this is important to fully understand the function of MANF in the neocortex. Related to this, it will be necessary to speculate the potential implication/limitation of the straight knockout (vs. conditional knockout selectively in the neocortex) in analyzing MANF function. For example, whether neocortical interneuron production/migration is affected, as this may contribute to the defects in cortical thickness, neurite outgrowth and neuronal differentiation. Also, do the knockout mice develop normally in general, e.g. brain/body weight ratio?

Minor points:

1. The authors need to clarify the use of NSC vs. NPC, as it is unclear whether they refer to the same or different cells.

2. It would be good to include the introduction of UPR, especially the markers of UPR used in the study.
